# Cinnamon Extract Enhances Glucose Uptake in 3T3-L1 Adipocytes and C2C12 Myocytes by Inducing LKB1-AMP-Activated Protein Kinase Signaling

**DOI:** 10.1371/journal.pone.0087894

**Published:** 2014-02-14

**Authors:** Yan Shen, Natsumi Honma, Katsuya Kobayashi, Liu Nan Jia, Takashi Hosono, Kazutoshi Shindo, Toyohiko Ariga, Taiichiro Seki

**Affiliations:** 1 Department of Chemistry and Life Science, Nihon University College of Bioresource Sciences, Nihon University Graduate School of Bioresource Sciences, Kanagawa, Japan; 2 School of Pharmacy, Nihon University, Chiba, Japan; 3 Department of Food and Nutrition, Japan Women’s University, Tokyo, Japan; Virginia Tech, United States of America

## Abstract

We previously demonstrated that cinnamon extract (CE) ameliorates type 1 diabetes induced by streptozotocin in rats through the up-regulation of glucose transporter 4 (GLUT4) translocation in both muscle and adipose tissues. This present study was aimed at clarifying the detailed mechanism(s) with which CE increases the glucose uptake *in vivo* and in cell culture systems using 3T3-L1 adipocytes and C2C12 myotubes *in vitro*. Specific inhibitors of key enzymes in insulin signaling and AMP-activated protein kinase (AMPK) signaling pathways, as well as small interference RNA, were used to examine the role of these kinases in the CE-induced glucose uptake. The results showed that CE stimulated the phosphorylation of AMPK and acetyl-CoA carboxylase. An AMPK inhibitor and LKB1 siRNA blocked the CE-induced glucose uptake. We also found for the first time that insulin suppressed AMPK activation in the adipocyte. To investigate the effect of CE on type 2 diabetes *in vivo*, we further performed oral glucose tolerance tests and insulin tolerance tests in type 2 diabetes model rats administered with CE. The CE improved glucose tolerance in oral glucose tolerance tests, but not insulin sensitivity in insulin tolerance test. In summary, these results indicate that CE ameliorates type 2 diabetes by inducing GLUT4 translocation *via* the AMPK signaling pathway. We also found insulin antagonistically regulates the activation of AMPK.

## Introduction

Diabetes mellitus is a common chronic disease in which a patient has persistent hyperglycemia. A number of epidemiologic studies have shown that as of year 2000, at least 171 million people worldwide have diabetes; and the number is estimated to be 2.8% in the world of the population [1]. Controlling the blood sugar concentration as close as possible to normal is very important to prevent the complication of diabetes. Thus therapeutic agents that ameliorate insulin resistance or stimulate the secretion of insulin and several kinds of insulin preparations for direct injection have received considerable attention. In recent years, several insulin-sensitizing agents have been developed that improve hyperglycemia through the activation of AMP-activated protein kinase (AMPK) [2], [3].

AMPK is highly conserved from yeast to plants and animals, and it plays a key role in the regulation of energy metabolism [4]. The activation of AMPK induces the translocation of glucose transporter 4 (GLUT4) to the plasma membrane [5], and several studies have demonstrated that AMPK and its signaling pathway are potential molecular targets in the development of drugs for the treatment of type 2 diabetes and obesity [6]–[9]. http://en.wikipedia.org/wiki/Diabetes_mellitus - cite_note-Wild2004-1#cite_note-Wild2004-1.

Cinnamon is an important component in Chinese medicine. Preparations made from the bark of trees of the genus *Cinnamomum* have been prescribed for more than 2000 years in China, and the first record for their use as Chinese medicine appeared in Shen-Nong’s Herbal [10]. There have been a number of *in vitro* and *in vivo* studies showing that cinnamon improves both insulin resistance and glucose metabolism [11]–[27]. However, the detailed mechanism of this anti-diabetic activity has not yet been clarified and is still controversial. Our previous study demonstrated that oral administration of a hot-water extract of cinnamon up-regulates mitochondrial uncoupling protein-1 (UCP-1) and enhances the production and translocation of GLUT4 in muscle and, at least, the translocation of GLUT4 in adipose tissues [27]. In this present study, we investigated the detailed molecular basis underlying the anti-diabetic effect of cinnamon by focusing on the insulin- and/or AMPK-signal pathways mediating glucose uptake in 3T3-L1 adipocytes and C2C12 myotubes. To investigate the effect of CE on type 2 diabetes *in vivo*, we further performed oral glucose tolerance tests and insulin tolerance tests in type 2 diabetes model rats administered with CE.

## Materials and Methods

### Preparation of Cinnamon Extract

Cinnamon (*Cinnamomum zeylanicum*) was a gift from House Foods Corporation (Tokyo). The cinnamon sticks (250 g) were soaked into 2,500 ml of water for 24 h at room temperature and then heated for 30 min at 100°C. The cinnamon extract was lyophilized and stored at −20°C until used.

### Reagents

The following primary antibodies against Akt, acetyl-CoA carboxylase (ACC), AMPKα, LKB1, p-Akt (Ser^473^), p-ACC (Ser^79^), p-AMPKα (Thr^172^), and p-LKB1 (Ser^428^) were purchased from Cell Signaling Technology (MA, USA). Anti-GLUT4 was from EMD Chemicals Inc. (Damstadt, Germany); and anti-E-cadherin, from Santa Cruz Biotechnology, Inc. (TX, USA). Anti-β-actin antibody was purchased from Sigma-Aldrich (MO, USA). Horseradish peroxidase-conjugated secondary antibodies were purchased from Jackson ImmunoResearch Laboratories (PA, USA). Insulin and compound C were obtained from Sigma-Aldrich and tyrphostin AG 1024 came from Alexis Biochemicals (CA, USA).

### Cell Culture

3T3-L1 fibroblasts (RIKEN BRC through the National Bio-Resource Project of the MEXT, Japan) were cultured and induced to differentiate into adipocytes by the method described previously [28]. Lipid droplets that appeared in the mature adipocytes were assayed colorimetrically after fixation with formaldehyde and stained with 0.5% Oil-Red O as described by Ramírez-Zacarías *et al.* [29]. C2C12 myoblasts (RIKEN BRC) were cultured in growth medium (DMEM with 4.5 g/l glucose, 4 mM glutamine, and 10% vol/vol fetal bovine serum). After 90% confluence had been reached the medium was changed to the differentiation medium (DMEM with 4.5 g/l glucose, 4 mM glutamine and 2% vol/vol fetal bovine serum) to stimulate myotube formation. The myoblasts were cultured in the differentiation medium for 8 days prior to the experiment.

### Measurement of Glucose Uptake

Glucose uptake was measured by the method described by Ragolia *et al.* [30]. Briefly, 3T3-L1 adipocytes and C2C12 myocytes cultured in 60-mm dishes (Becton Dickinson, NJ, USA) were serum-starved in DMEM for 16 h, and the cells were then washed 3 times with PBS (pH 7.4) and subsequently incubated for 30 min in the following medium: DMEM alone, DMEM containing either 100 nM insulin or 30 µg/ml CE, or the combination of both insulin and CE. Then, 0.5 mM 2-deoxy-D-[2,6–^3^H] glucose (1.5 µCi/well, Moravek Biochemicals, CA, USA) was added to the cells, which were then incubated for 15 min. Finally, the cells in triplicate were washed 4 times with PBS containing 0.3 mM phloretin and thereafter lysed in 1 ml of 1N NaOH for scintillation counting.

### Western Blot Analysis

Whole-cell lysates and plasma membrane fractions were subjected to SDS-PAGE, and the proteins that had migrated were electrically transferred to a polyvinylidene fluoride (PVDF) microporous membrane (Millipore Corporation, MA, USA) for Western blotting. The membrane was incubated at 4°C for 18 h with antibodies against the following proteins: p-Akt, Akt, p-AMPKα, AMPKα, p-ACC, ACC, p-LKB1, LKB1, p-PDK1, PDK1, GLUT4 (1∶1000), E-cadherin (1∶200), and β-actin (1∶2000). After the incubation, anti-mouse IgG horseradish peroxidase-conjugate (HRP, 1∶2000, for β-actin) or anti-rabbit IgG HRP (1∶2000, for p-Akt, Akt, p-AMPKα, AMPKα, p-ACC, ACC, p-LKB1, LKB1, p-PDK1, PDK1, and GLUT4) was added; and the membrane was incubated for 30 min at room temperature. The antigenic proteins on the membrane were visualized by chemiluminescence by use of a Lumi-LightPLUS Western blotting kit (Roche Diagnostics Co., Basel, Switzerland), and the images were evaluated by using an Image Analyzer LAS-4000 (Fujifilm Co. Tokyo, Japan).

### Isolation of Plasma Membranes from 3T3-L1 Adipocytes

3T3-L1 adipocytes were homogenized by sonication for 5 min at 3 kHz/130 W (UCD-130TM, Cosmo Bio Co., Tokyo, Japan) in ice-cold HES buffer (0.02 M HEPES, 0.25 M sucrose, and 2 mM EGTA; pH 7.4) and centrifuged at 700×*g* for 7 min to remove unhomogenized cellular debris and nuclei from the homogenate. The harvested supernatant was further centrifuged at 760×*g* for 10 min to remove mitochondria. It was recentrifuged at 35,000×*g* for 60 min, and the resulting pellet was used as the plasma membrane fraction of the adipocytes. The supernatant was used as the cytosol fraction [31]. These membrane and cytosol fractions were subjected to Western blotting to detect GLUT4 and E-cadherin, respectively. The amounts of protein in the cytosolic fraction and membrane pellet were quantified by using Protein Assay Dye Reagent (Bio-Rad Laboratories, Inc., PA, USA).

### RNA Isolation and Real-time PCR

Total RNA was extracted from 3T3-L1 adipocytes and C2C12 myotubes by use of Isogen reagent (Wako Pure Chemical Industries). Real-time reverse transcription-PCR was performed by the fluorescent dye SYBR Green I method using SYBR Premix Ex Taq and Perfect Real Time (Takara Bio, Shiga, Japan) with a StepOne PCR system (Life Technologies, CA, USA). The primers used in this study were designed based on GenBank information (Stk11, NM_011492) and synthesized by Life Technologies. The PCR primers used for LKB1 were 5′-CAC ACT TTA CAA CAT CAC CA-3′ and 5′-CTC ATA CTC CAA CAT CCC TC-3′ `.

### Silencing of LKB1 Gene Expression by RNA Interference

LKB1 siRNA (Stealth RNAi, Life Technologies) and control siRNA (Life Technologies) were used. The 3T3-L1 adipocytes or C2C12 myotubes cultured in 60-mm dishes (Becton Dickinson) were transfected with a 0.5 µM concentration of either siRNA by using the transfection reagent Lipofectamine 2000 (Life Technologies). Prior to transfection, 12.5 µl of siRNA and 5 µl of transfection reagent were diluted with 500 µl of serum-free medium in separate tubes at first, and then the diluted siRNA and Lipofectamine were mixed together. The mixtures were incubated for 10 min at room temperature and then added dropwise to each culture well. Four hours after transfection, the medium was exchanged for fresh medium. The cells were cultured for 24 h prior to the experiment. The efficiency of LKB1 silencing was monitored by both quantitative reverse transcription-PCR and Western blotting.

### Animal Experiment

Four-week-old male Otsuka Long-Evans Tokushima Fatty (OLETF) rat, a model of type 2 diabetes mellitus, and Long-Evans Tokushima Otsuka (LETO), a non-diabetic normal control, were provided by Otsuka Pharmaceutical Co., Ltd. (Tokushima Research Institute, Tokushima, Japan) and housed individually in a stainless steel wire-bottomed cage in a temperature-controlled room at 22–23°C with a 12 h photoperiod. OLETF and LETO rats were kept on AIN-93G diet (Clea Japan Inc., Tokyo, Japan) and water *ad libitum*. After 1 week of acclimation, rats were divided into 2 groups with matched body weight; *i.e.,* 1) Control group without cinnamon (LETO, OLETF); 2) cinnamon treated group (LETO+CE, OLETF+CE). All experiments were performed in accordance with the National Institutes of Health Guide for the Care and Use of Laboratory Animals, and were approved by the Nihon University Animal Care and Use Committee.

### Oral Glucose Tolerance Test and Insulin Tolerance Test

OLETF and LETO rats were orally administered CE (100 mg/kg. b.w./day) for 15 weeks. For the glucose tolerance test, rats were fasted for 16 h followed by oral administration of glucose (1.2 g/kg b.w.). The blood-glucose concentration was measured by a DEXTER-Z II (Bayer Medical Co., Ltd., Leverkusen, Germany) using a blood collected from a tail vein. For insulin tolerance test, rats were fasted for 4 h, then the insulin (0.75 U/kg b.w.) was administered intraperitoneally followed by the measurement of blood glucose concentration as described above. Plasma insulin concentration was assayed by using an ELISA kit (Shibayagi Co., Ltd., Gunma, Japan).

### Statistical Analysis

The Student’s t test was used in comparing two sets of data. Statistical differences among more than three groups were determined using one-way analysis of variance (ANOVA) with repeated measures followed by Bonferroni multiple comparison tests. Statistical analysis was performed by using GraphPad Prism software v6.0 (GraphPad Software, CA, USA). A value of *p*<0.05 was considered statistically significant. All data are expressed as mean ± S.E.

## Results

### CE Stimulated Glucose Uptake and GLUT4 Translocation in 3T3-L1 Adipocytes

**Figure 1:**
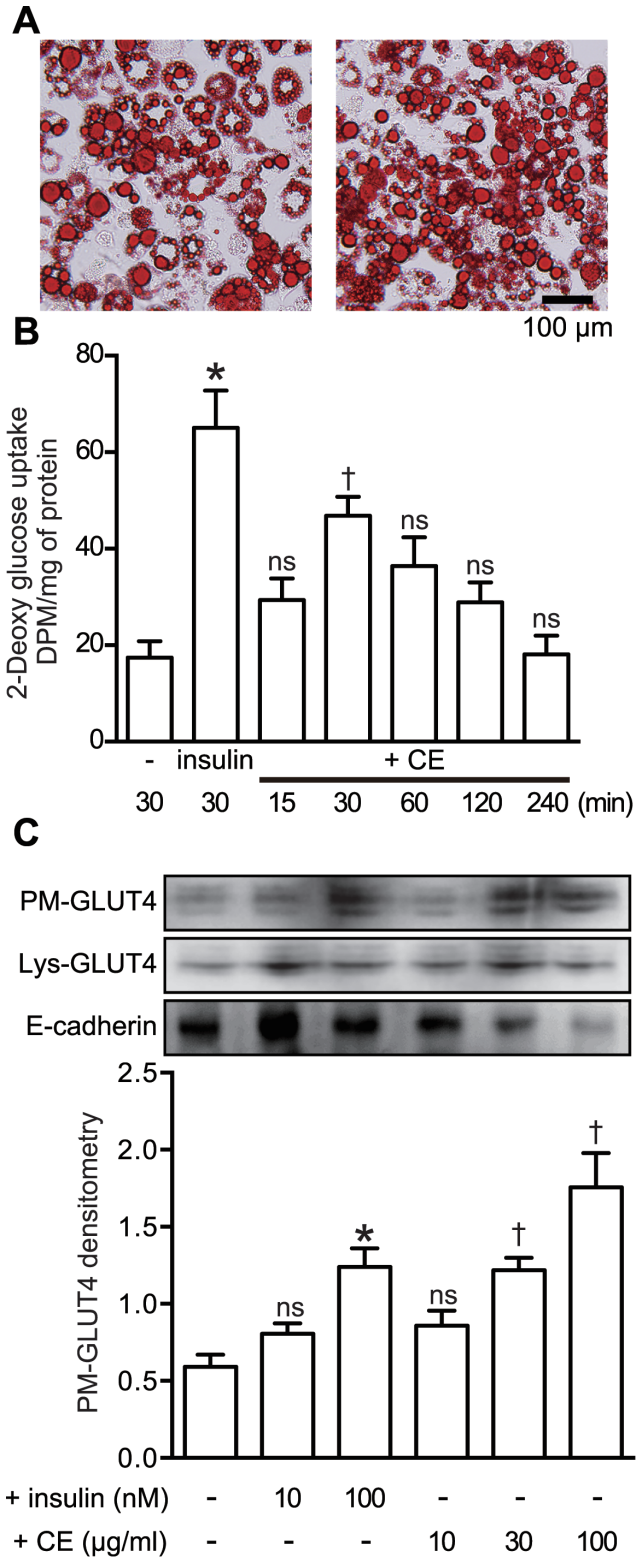
Effect of CE on glucose uptake and translocation of GLUT4 in 3T3-L1 adipocytes. A: Appearance of oil red O-stained 3T3-L1 adipocytes differentiated from mouse 3T3-L1 fibroblasts. The photomicrographs show typical adipocytes found in different fields of observation. B: 3T3-L1 adipocytes were serum-starved for 16 h in DMEM, and then challenged with CE (30 µg/ml) for 15–240 min or with insulin (100 nM) for 30 min, and thereafter assayed for the uptake of 2-deoxyglucose by the cells, as described in the Materials and Methods section (n = 3). C: GLUT4 expression in the adipocytes stimulated with either insulin or CE for 30 min. The adipocytes were retrieved after the stimulation, and the plasma membrane fraction prepared was analyzed by Western blotting as described in the text (n = 4). Values are the means ± S.E. *, *p*<0.05 between insulin and control; †, *p*<0.05 between CE and control (Bonferroni test). PM, plasma membrane fraction; lys, lysate of the 3T3-L1 adipocytes.

To clarify the anti-diabetic effect of CE, we first examined the effect of CE on glucose uptake and translocation of GLUT4 to the plasma membrane in differentiated 3T3-L1 adipocytes. Oil red O-stained 3T3-L1 adipocytes that differentiated from the 3T3 fibroblasts are shown in Fig. 1A. As shown in Fig. 1B, 30 µg/ml CE significantly increased the 2-deoxyglucose uptake into the 3T3-L1 adipocytes in a treatment time-dependent manner, with peak uptake at 30 min. Insulin (100 nM), which we employed as a positive control, also potently stimulated the uptake of 2-deoxyglucose. Consistent with the glucose uptake, significant translocation of GLUT4 was also observed in both insulin- and CE-treated adipocytes (Fig. 1C). The concentration of CE we used in this study did not show any cytotoxicity toward 3T3-L1 adipocytes, as assessed by the MTT assay (data not shown).

### CE Caused Phosphorylation of AMPK in 3T3-L1 Adipocytes

**Figure 2:**
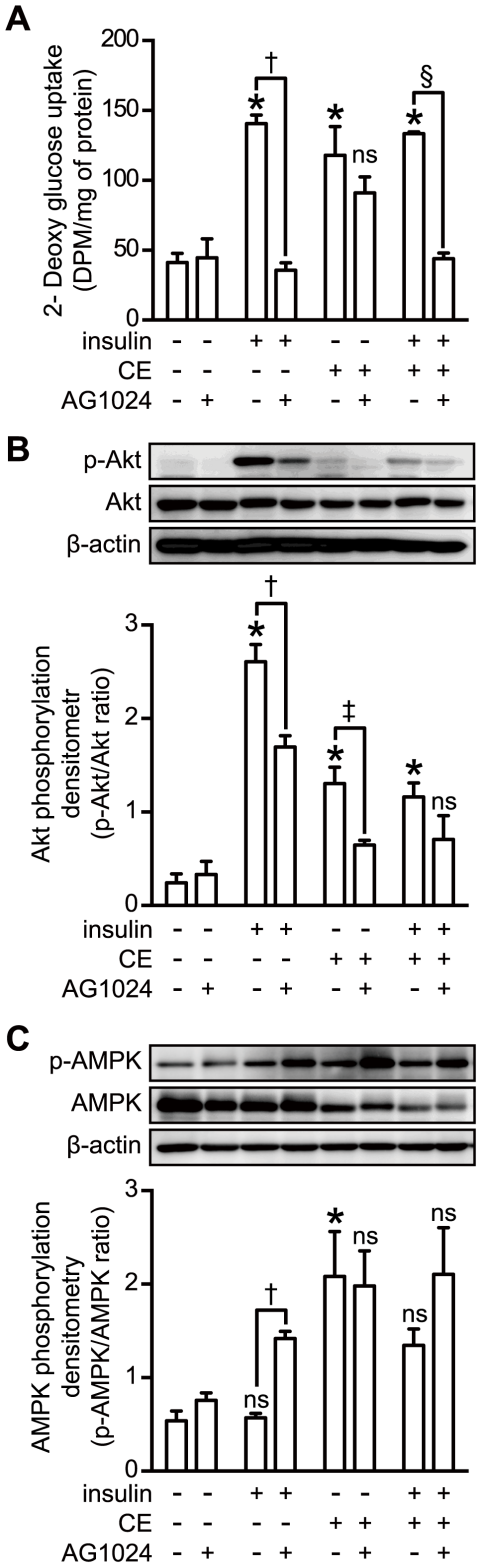
Effect of inhibitors of insulin signaling kinases on CE-mediated glucose uptake and phosphorylation of AMPK. 3T3-L1 adipocytes were serum-starved for 16 h. For the inhibitor treatment, the cells were pretreated with 60 µM AG1024 for 1 h. The medium was then changed to fresh medium containing 100 nM insulin or 30 µg/ml CE and thereafter stimulated for another 30 min. A: Glucose uptake was measured as described in Materials and Methods section. Each value represents the mean ± S.E. of 3 different experiments. B: Cell lysates were analyzed by Western blotting using anti-phospho-Akt and anti-Akt antibodies. C: Cell lysates were analyzed by Western blotting using anti-phospho-AMPK anti-AMPK antibodies. The data represent the mean ± S.E. of 5 different experiments. *, *p*<0.05 *vs* control; †, *p*<0.05 *vs* insulin; ‡, *p*<0.05 *vs* CE; §, *p*<0.05 *vs* insulin+CE; ns: indicates no significant difference (Bonferroni test).

To investigate the molecular mechanism underlying CE-stimulated GLUT4 translocation and glucose uptake, we studied key kinases involved in the insulin signaling pathway. Insulin-induced glucose uptake was completely cancelled by pretreatment of the adipocytes with the insulin receptor inhibitor AG1024; however, AG1024 could not block the effect of CE (Fig. 2A). Intriguingly, insulin or CE increased the phosphorylation of Akt at its Ser^473^, and this increase was significantly blocked by AG1024 (Fig. 2B). These results suggest that insulin receptor-Akt signaling was not a major pathway involved in the CE-stimulated glucose uptake. Then we evaluated the contribution of AMPK to the CE-stimulated glucose uptake. CE potently increased the level of phosphorylated AMPK (Fig. 2C). AG1024 did not show any effect on this CE-induced AMPK phosphorylation, but it significantly increased the insulin-mediated phosphorylation of AMPK. Insulin seemed to have little direct effect on the phosphorylation of AMPK in the adipocytes and this inefficacy was abrogated by pre-incubation of the cells with AG1024. Taken together, these results indicate that CE up-regulated glucose uptake through the phosphorylation of AMPK pathway.

### LKB1, AMPK, and ACC are Activated by CE

**Figure 3:**
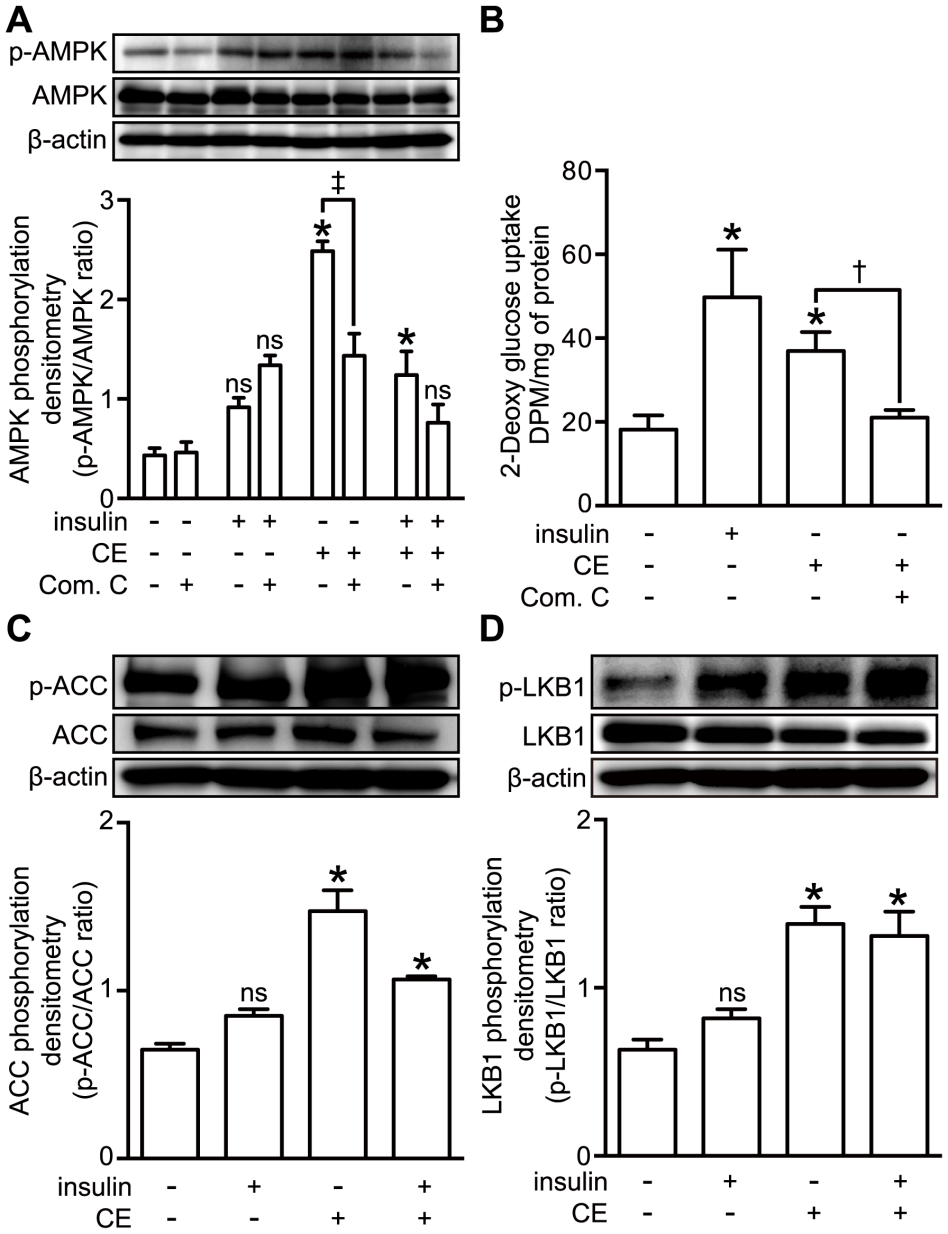
Effect of AMPK inhibitor on CE-stimulated glucose uptake and ACC, LKB1 phosphorylation in 3T3-L1 adipocytes. 3T3-L1 adipocytes were serum-starved for 16 h. For the inhibitor treatment, cells were pretreated with 200 µM Compound C (Com.C), an inhibitor of AMPK, for 20 min, after which the medium was changed to fresh medium containing 100 nM insulin or 30 µg/ml CE. The cells were then incubated for another 30 min. A: Cell lysates were analyzed by Western blotting using anti-phospho-AMPK and anti-AMPK antibodies. The data represent the mean ± S.E. of 5 different experiment *: *p*<0.05 *vs* control; ‡: *p*<0.05 *vs* CE; ns: indicates no significant difference (Bonferroni test). B: Glucose uptake was measured as described in the Materials and Methods section. Each value represents the mean ± S.E. of 3 different experiments. *: *p*<0.05 vs control; †: *p*<0.05 *vs* CE; ns: indicates no significant difference (Bonferroni test). C, D: Cell lysates were analyzed by Western blotting using anti-phospho-ACC, anti-phospho- LKB1, anti-ACC, and anti-LKB1 antibodies. The data represent the mean ± S.E. of 5 different experiments. *: *p*<0.05 *vs* control; ns: indicates no significant difference (Bonferroni test).

To corroborate the role of AMPK in CE-mediated glucose uptake, we examined the effect of an AMPK inhibitor. First, 3T3-L1 adipocytes were pre-treated with compound C (200 µM, Com.C), an AMPK inhibitor, to test the effect of it on the phosphorylation of AMPK and uptake of 2-deoxyglucose by the adipocytes stimulated with CE (30 µg/ml). The CE-induced phosphorylation of AMPK was significantly decreased by pre-incubation of the adipocytes with Com.C (Fig. 3A). The CE-stimulated glucose uptake was also attenuated by Com.C (Fig. 3B), indicating the involvement of the AMPK pathway in the CE-induced glucose uptake. Next, we examined the phosphorylation of factors located downstream and upstream of AMPK. As shown in Fig. 3C, the increase in phosphorylated AMPK was accompanied by that of phosphorylated ACC (Ser^79^), which enzyme is located downstream of AMPK. The phosphorylation of LKB1, which acts upstream of AMPK, was also increased by CE treatment (Fig. 3D). Therefore, these findings show that CE activated the LKB1-AMPK-ACC signaling cascade, eventually leading to increased glucose uptake.

### CE Stimulated AMPK through the LKB1 Pathway in C2C12 Myotubes

**Figure 4:**
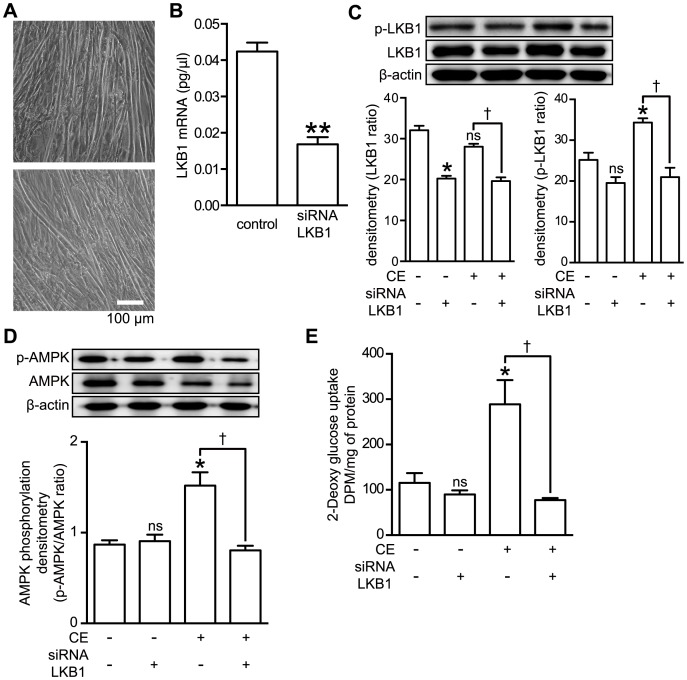
Effect of LKB1 knockdown on AMPK phosphorylation and CE-stimulated glucose uptake. Differentiated C2C12 cells (A) were transfected with LKB1 siRNA for 24 h. Two different fields of observation are shown. B: Quantitative real-time PCR was used to determine the mRNA expression levels of LKB1 by the C2C12 cells that had been treated with LKB1 siRNA. The data represent the mean ± S.E. of 3 different experiments. **: *p*<0.01 (Student’s t test). C: Effect of siRNA duplexes designed to reduce LKB1 expression on the LKB1 protein levels and on the phosphorylation of LKB1 in C2C12 myotube cells examined by Western blotting. These silencing effects were observed even in the culture at 24 h after the transfection. The data represent the mean ± S.E. of 4 different experiments. *: *p*<0.05 *vs* control; †: *p*<0.05 *vs* CE; ns: indicates no significant difference (Bonferroni test). D: CE (30 µg/ml) was added or not to LKB1 siRNA-transfected cells or negative control siRNA-transfected cells, which were then incubated for 30 min and thereafter harvested and analyzed by Western blotting using anti-phospho-AMPK, and anti-AMPK antibodies. The data represent the mean ± S.E. of 3 different experiments. *: *p*<0.05 *vs* control; †: *p*<0.05 *vs* CE; ns: indicates no significant difference (Bonferroni test). E: Differentiated C2C12 cells were transfected or not with LKB1 siRNA or negative control siRNA for 24 h and then treated or not with CE (30 µg/ml) for 30 min. The glucose uptake was measured by the method as described in the Materials and Methods section. The data represent the mean ± S.E. of 3 different experiments. *: *p*<0.05 *vs* control; †: *p*<0.05 *vs* CE; ns: indicates no significant difference (Bonferroni test).

In addition to the white adipose tissue, muscle plays physiologically important roles in the glucose metabolism. To confirm further the function of LKB1 in the muscle cells, LKB1 siRNA was used to transiently transfect C2C12 muscle cells. The appearance of the differentiated C2C12 cells used in this study is shown in Fig. 4A. The 2 photomicrographs, taken at different fields of a culture, show the typical myotube phenotype. The LKB1 mRNA level and the CE-induced phosphorylation of LKB1 at its Ser^428^ were decreased by approximately 60% and 40%, respectively, with siRNA pools specific for the LKB1 gene (siLKB1) in comparison with the respective values for the cells transfected with the RNAi negative control (Fig. 4B and C).

Knockdown of LKB1 attenuated the CE-induced phosphorylation of AMPK (Fig. 4D). The knockdown of LKB1 by siRNA also suppressed CE-stimulated glucose uptake (Fig. 4E). These results suggest that AMPK mediated the effect of CE on signaling pathway in an LKB1-AMPK-dependent manner.

### CE-administration Improved Glucose Tolerance in Type 2 Diabetes Model Rats

**Figure 5:**
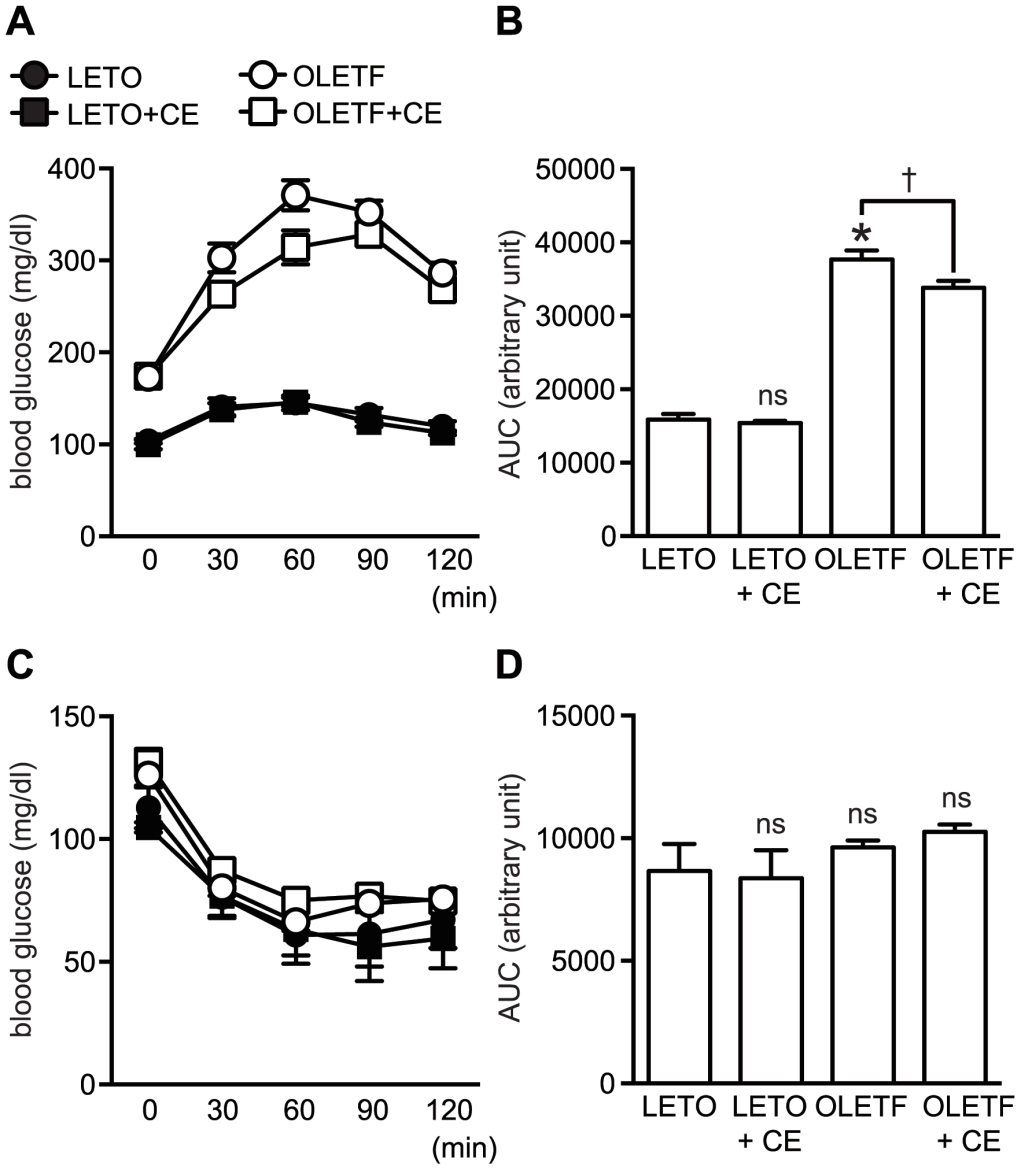
Effect of CE administration on glucose and insulin intolerance in type 2 diabetic rats. A: For oral glucose tolerance test (OGTT), the rats were fasted for 6 h prior to performing the test. Glucose (1.2 g/kg b.w.) was orally administered and the blood-glucose concentration was measured by using the blood obtained from a tail vein at the indicated time points after the oral glucose loading. B: The area under the curve (AUC) of the OGTT for A. C: For insulin tolerance test (ITT), rats were fasted for 4 h. Then insulin (0.75 U/kg b.w.) was intraperitoneally injected and the blood-glucose concentration was measured as described above. D: The area under the curve (AUC) of the ITT for C. Data were analyzed by Bonferroni test. (n = 5–10 for each group, mean ± S.E.). *: *p*<0.05 *vs* LETO; †: *p*<0.05 *vs* OLETF; ns: indicates no significant difference.

To determine the effect of CE on type 2 diabetes, we performed the oral glucose tolerance test (OGTT) and insulin tolerance test (ITT) in type 2 diabetes model rats *in vivo* (Fig. 5). CE administration did not show any effect on body weight (Table 1). In OGTT, CE-treated OLETF rats had lower blood glucose concentrations than that of vehicle-treated OLETF rats (Fig. 5A, 5B) indicating that the improved glucose tolerance in type 2 diabetes model rats. On the other hand, CE did not show any effect on the blood glucose concentration of LETO rats.

**Table 1:** Effect of CE administration on body weight and plasma insulin concentrations.

Group	Body weight (g)	Insulin (ng/ml)
LETO	460.14±13.95	7.99±0.90
LETO+CE	440.88±9.87	9.05±0.78
OLETF	655.72±7.21	27.79±4.40
OLETF+CE	655.44±7.85	30.04±4.66

Values are mean ± S.E. of 7 different rats.

ITT revealed that insulin sensitivity was not influenced by CE treatment in both LETO and OLETF rats (Fig. 5C, 5D).

## Discussion

The principal finding of this study is that a hot-water extract of cinnamon (CE) had a metabolic effect on adipocytes and skeletal muscles. Specifically, we demonstrated for the first time that the LKB1-AMPK signaling pathway is involved in CE-stimulated glucose metabolism and that CE improves glucose tolerance *in vivo*.

AMPK is a heterotrimeric complex composed of a catalytic subunit α and regulatory β and γ subunits [32]. AMPK is activated by phosphorylation of its Thr^172^ residue located in the activation-loop of the α subunit [33]–[35]. It has been reported that AMPK is activated synergistically by AMP and the phosphorylation of this Thr^172^ for insulin-independent glucose transport [36]. Activation of AMPK induces the recruitment of GLUT4 to the plasma membrane, resulting in up-regulated glucose uptake [5], [37]. In this study, we demonstrated that CE stimulated the translocation of GLUT4 to the plasma membrane and increased glucose uptake in a concentration and time-dependent manner (Fig. 1B and C). To clarify the relationship between CE-activated glucose metabolism and insulin-independent glucose transport, we used an insulin receptor inhibitor, tyrphostin AG 1024 (AG1024) and an AMPK inhibitor, compound C, in addition to transiently LKB1 knock-downed cells, which have been proven to lack insulin or AMPK-signaling. In fact, we confirmed that CE stimulated glucose uptake was cancelled by inhibition of AMPK and its upstream kinase, the LKB1 (Figs. 3B and 4E). These results strongly suggest that activation of LKB1-AMPK signaling by CE in both adipocyte and muscle cell up-regulates glucose uptake into the cells, but not insulin signaling.

CE caused the phosphorylation of Akt, a kinase acting downstream of the insulin receptor, but little is known about the cross talk between AMPK and insulin signaling. In this study, insulin did not show any additive or synergistic effect with CE in respect to glucose uptake. Actually, insulin signaling was very weakly influenced by CE treatment in comparison with the effect of CE on AMPK signaling. Intriguingly, inhibition of insulin receptors by AG1024 caused an increase in the AMPK activity in the presence of insulin (Fig. 2C), thus suggesting negative regulation of AMPK activity by insulin signaling. It has been reported that insulin inhibits AMPK activity *via* the PI3K pathway in ischemic cardiac muscle [38]–[39]. To the best of our knowledge, this is the first report to demonstrate the antagonistic effect between insulin and AMPK signaling in adipocytes.

Berberine, an effective compound of Chinese traditional herbal medicine, and *S*-allyl cysteine in garlic have been reported to ameliorate diabetes and obesity *via* stimulation of AMPK activity; however, the mechanism for AMPK phosphorylation or its upstream signaling remains unclear [40]–[42]. The tumor suppressor LKB1, a protein ubiquitously expressed by mammalian cells, has been demonstrated to activate AMPK [35], [43]–[47]. It has been reported that LKB1 is activated by two putative kinases; *i.e.,* STE20-related adaptor protein (STRAD) and mouse protein 25 (MO25) [48]. STRAD possesses a domain with high sequence homology to protein kinases but it lacks the key catalytic residues required for catalysis and has therefore been classified a ‘pseudokinase’. MO25 has no sequence homology with other proteins in the database [49]. LKB1 has been shown to phosphorylate and activates AMPK in response to metformin and energy depletion in cell lines [35], [43] as well as in mouse liver and muscle [50], [51]. Although there are many papers reporting the activation of AMPK by natural compounds, the detailed mechanism(s) for the activation still remains unclear. In this study, we speculate that CE promotes the formation of LKB1 complex with STRAD and MO25 to activate AMPK by the phosphorylation of Thr^172^.

Finally, we observed that CE improved glucose intolerance but not insulin resistance in type 2 diabetic rats (Fig. 5). This observation is consistent with the anti-diabetic effect of CE on insulin-uncontrolled type 1 diabetic rats which we reported previously [27]. In our studies, we identified two compounds in the CE by high-performance liquid chromatography analyses; the CE preparation contained cinnamaldehyde (8.5 µg/mg CE) and cinnamyl alcohol (3.6 µg/mg CE) [27]. Since both cinnamaldehyde and cinnamyl alcohol potently stimulated glucose uptake in 3T3-L1 adipocytes in our studies (data not shown), these compounds are thought to be, at least in partly, responsible for the effect of CE.

In conclusion, the current study demonstrated for the first time that CE stimulates LKB1-mediated phosphorylation of AMPK to increase glucose uptake in 3T3-L1 adipocytes and C2C12 myotubes. Since insulin resistance has been linked with the progressive development of type 2 diabetes mellitus, natural compounds that stimulate AMPK activity should have a significant clinical impact on type 2 diabetes mellitus.
